# A retrospective study on the clinical and molecular outcomes of calpainopathy in a Turkish patient cohort

**DOI:** 10.55730/1300-0144.5769

**Published:** 2023-12-18

**Authors:** İzem Olcay ŞAHİN, Emine KARATAŞ, Mikail DEMİR, Büşra TAN, Hüseyin PER, Yusuf ÖZKUL, Munis DÜNDAR

**Affiliations:** 1 Department of Medical Genetics, Faculty of Medicine, Erciyes University, Kayseri, Turkiye; 2 Department of Pediatric Neurology, Faculty of Medicine, Children’s Hospital, Erciyes University, Kayseri, Turkiye

**Keywords:** LGMDR1, LGMD2A, LGMDD1, NGS, variants

## Abstract

**Background and aim:**

Calpainopathy, also known as limb-girdle muscular dystrophy recessive type 1, is a progressive muscle disorder that impacts the muscles around the hips and shoulders. The disease is caused by defects in the *CAPN3* gene and can be inherited in both recessive and dominant forms. In this retrospective study, we aimed to evaluate the clinical and molecular results of our patients with calpainopathy and to examine the *CAPN3* variants in Turkish and global populations.

**Materials and methods:**

Molecular analyses were performed using the next-generation sequencing (NGS) method. *CAPN3* variants were identified through the examination of various databases.

**Results:**

In this retrospective study, the cohort consisted of seven patients exhibiting the *CAPN3* (NM_000070.3) mutation and a phenotype compatible with calpainopathy at a single center in Türkiye. All patients displayed high CK levels and muscle weakness. We report a novel missense c.2437G>A variant that causes the autosomal dominant form of calpainopathy. Interestingly, the muscle biopsy report for the patient with the novel mutation indicated sarcoglycan deficiency. Molecular findings for the remaining individuals in the cohort included a compound heterozygous variant (frameshift and missense), one homozygous nonsense, one homozygous intronic deletion, and three homozygous missense variants. The most common variant in the Turkish population was c.550del. In both populations, pathogenic variants were most frequently located in exon 21, according to exon length. Variants were stochastically distributed based on consequences in CAPN3 domains.

**Conclusion:**

Therefore, the NGS method proves highly effective in diagnosing rare diseases characterized by clinical heterogeneity. Assessing variants based on ethnicity holds significance in the development of precise therapies.

## 1. Introduction

Limb-girdle muscular dystrophy recessive type 1 (LGMDR1) is a progressive disease characterized by defects in the Calpain-3 (*CAPN3*) gene (NM_000070.3; NP_000061.1), leading to muscle weakness in the proximal parts of the upper and lower extremities [1]. The prevalence of LGMDR1 ranges from 1 to 9 per 100,000 people, constituting approximately 30% of all limb-girdle muscular dystrophies (LGMDs) [1] 1. In Türkiye, this percentage increases to 40%–50% [2,3]. The onset of LGMDR1 typically occurs in adolescence, spanning a broad age range from childhood to adulthood. Loss of ambulation typically occurs at a mean of 17.3 years after the onset of the disease 2. LGMDR1, also referred to as calpainopathy, follows an autosomal recessive inheritance pattern, although it can also be inherited in an autosomal dominant manner, as seen in limb-girdle muscular dystrophy dominant type 1 (LGMDD1) [4].

In LGMDR1, the muscles of the limb-girdle and trunk are affected, with notable involvement of gluteus maximus and thigh adductor [5]. Alongside symmetric muscle weakness, patients exhibit clinical findings such as scapular wing, joint contracture, Achilles tendon shortening, scoliosis, and high CK levels [5]. The majority of these variants arise from single nucleotide alterations in the coding region, with most being missense changes 3. It is worth noting that the full-length transcript (ENST00000397163.8) of the *CAPN3* gene, is exclusively found in skeletal muscle and is located at 15q15.1 [6].

The *CAPN3* gene consists of 24 exons, and its protein product contains 821 amino acids. Similar to other classical calpains, CAPN3 consists of a large subunit with four domains (I-IV). Unlike the other calpains, CAPN3 lacks small subunits [7] and contains specific sequences: N-terminus (NS), insertion sequence (IS) 1, and IS 2 [8]. Domain I contains NS, which is effective in the formation of the homotrimer structure [9], while Domain II, which is a conserved cysteine protease domain, contains protease core domain (PC) 1 and PC 2, which provides protease activity [9]. The calpain-type sandwich domain (CBSW), also known as Domain III, is a critical site for structural changes in active CAPN3 [9]. Domain IV, also known as the Penta-EF-hand (PEF) domain, is responsible for Ca^2+^ binding and homodimerization of CAPN3 [9]. IS1, located within the PC2, is required for protease activity [10], while IS2, located between the CBSW and PEF domains, contains a nuclear translocation sequence [11].

Although our current understanding of the function of the CAPN3 protein is incomplete, relevant studies have shown that CAPN3 exhibits both proteolytic and nonproteolytic activities. CAPN3 plays a critical role in regulating sarcomere stability/integrity and muscle contraction by activating its substrates through its proteolytic property [12,13]. CAPN3 exerts its nonproteolytic activity by localizing and stabilizing the proteins that maintain Ca^2+^ homeostasis [14–16]. Additionally, it is a unique protein capable of recovering its protease activity after its autolytic dissociation through the intermolecular complementation process [17]. Despite the development of various treatment strategies, there is currently no definitive treatment for LGMDR1 [18].

In this retrospective study, our goal was to assess both the molecular and clinical outcomes of seven patients with calpainopathy at our center. Furthermore, we aimed to investigate variations in the molecular profile of CAPN3 in individuals with calpainopathy from Türkiye and around the world. This involved a comprehensive analysis of CAPN3 variants, drawing on an extensive review of the existing literature.

## 2. Methods

### 2.1. Patient recruitment

This retrospective study included seven patients who sought medical attention at Erciyes University Medical Faculty Hospital, Department of Medical Genetics, in Türkiye between January 2018 and December 2021. The patients were identified to have *CAPN3* mutations through molecular analysis. Clinical histories of the patients were obtained from medical records to the extent possible. Although muscle biopsy images were not available, the results obtained from the medical records were interpreted. The study received approval from the Erciyes University Non-Clinical Research Ethics Committee (approval no. 2022/111).

### 2.2. Molecular analysis

Molecular analyses for all patients were performed using next-generation sequencing (NGS) technology at the Erciyes University Department of Medical Genetics. To align with the preliminary diagnosis, patients were assessed using the Hereditary Disorder Panel, comprising 569 genes (refer to Appendix 1), including *CAPN3* (NM_000070.3). Exome sequencing of these genes was performed. DNA isolation from peripheral blood samples was achieved using the MagNA Pure LC DNA isolation kit (Roche Diagnostic GmbH, Mannheim, Germany) and the MagNA Pure LC 2.0 device (Roche Diagnostic Ltd., Rotkreuz, Switzerland) following the manufacturer’s instructions. The quantification of DNA concentration and enrichment of the library were conducted using a Qubit® 3.0 Fluorometer (Invitrogen, Life Technologies Holdings Pte Ltd, Malaysia). The size distribution of the library was assessed with the Agilent 2100 Bioanalyzer (Agilent Technologies, Waldbronn, Germany). The Hereditary Disorder Solution kit (SOPHiA GENETICS, Saint-Sulpice, Switzerland) was used for library preparation. DNA sequencing was performed on an Illumina NextSeq 500 instrument (Illumina, Inc., San Diego, CA, USA). Bioinformatic analysis was carried out via Sophia DDM v5.10.11.1 (SOPHiA GENETICS, Saint Sulpice, Switzerland). Variant interpretation followed the 2015 guidelines from the American College of Medical Genetics and Genomics (ACMG) [19], with only pathogenic, likely pathogenic, and variants of unknown significance being reported. Moreover, *CAPN3* gene-targeted Sanger sequencing was performed to validate the novel candidate mutation and analyze the family segregation pattern. Sequencing was carried out on the 3500 Genetic Analyzer instrument, and SeqScape Software v3.0 was used for data analysis.. Evaluation of changes adhered to the criteria outlined in the ACMG 2015 guidelines. Refer to Appendix 2 for the primer designed for Sanger sequencing.

### 2.3. In silico analysis of novel variant

The Clustal Omega program was employed to perform multiple alignments of CAPN3 protein sequences. Sequences from various species were obtained from the UniProt database. To assess the clinical significance of the novel variant, we utilized BayesDel-addAF, BayesDel-NoAF, LoFtool, CADD-Phred, ClinPred, PROVEAN, and MUTATION TASTER that we accessed through VEP [20], and the POLYPHEN-2 [21] tool.

To enhance our comprehension of the potential deleterious modification caused by the novel variant at the structural level, we performed a three-dimensional (3D) modeling study using UCSF Chimera v1.15 software. Utilizing the CAPN3 protein template derived from Alphafold v2.0, a 3D model was constructed.

### 2.4. Literature review and data extraction

Allele datasets for *CAPN3* variants associated with calpainopathy reported from Türkiye were obtained from the LOVD database and relevant literature. Globally reported variants were sourced from gnomAD and ClinVar. On the gnomAD database, only variants located in or within 75 base pairs of a coding exon were considered. Variants with a minor allele frequency (MAF) less than 0.01 were selected from the global dataset. Annotation of variants, including consequences, exon and intron numbers, clinical significance, etc., was obtained from Ensemble VEP, Varsome, and Franklin tools (NM_000070.3). The clinical significance of the variants was categorized as pathogenic and likely pathogenic, variants of unknown significance (VUS), and benign. Variant distribution was analyzed in terms of exon and intron numbers in Turkish and global patients. Since exons and introns have different lengths, the average number of variants per length was calculated to estimate the mean degree of risk. The percentages of variant consequences were determined. Additionally, we explored whether variants exhibited frequency in specific domains. Domains were identified using UniProt as a reference and categorized as catalytic, EF-hand 1, EF-hand 2, EF-hand 3, and EF-hand 4. The datasets were analyzed using Microsoft Excel.

## 3. Results

### 3.1. Clinical phenotype

We evaluated the clinical and molecular outcomes of seven affected patients from six families. Six of the patients were from Türkiye, and one was from Syria. Within our cohort, only Family 6 (F6) included two affected siblings; the remaining families had only one affected individual. A summary of the clinical status of the patients is provided in Table 1. The mean age of symptom onset for the patients was 12.86 years, ranging from 7 to 20 years. Muscle weakness in the proximal lower extremities was the initial symptom for all patients except for F6-II, who exhibited a wobbly gait. Patient F5 had undergone an Achilles tendon lengthening operation in childhood, and F6-I walked on tiptoes during childhood. With the exception of F6-II, all patients demonstrated a progression of muscle weakness over time. All patients, excluding F1, were ambulant at the time of the examination. Elevated CK levels were observed in all patients, significantly exceeding normal values. While contracture status information for F3 and F4 was unavailable, contracture was present in F1 and F6-I but not in F2, F5, or F6-II. Calf hypertrophy was noted in F3 and F4 but absent in F1, F2, or F6-II. The status of calf hypertrophy for F5 and F6-I could not be determined.

### 3.2. Histopathological evaluation

Based on muscle biopsy results, all patients exhibited dystrophic patterns, except for F2 and F3. In the muscle biopsies of F2 and F3, slightly altered fiber sizes were observed. Interestingly, CAPN3 levels were positive in F2, while dystrophin and β-dystroglycan levels were either partially or completely negative. In the case of F1’s muscle biopsy, there was a decrease in γ and δ sarcoglycan levels.

### 3.3. Molecular analysis results

**Figure 1:**
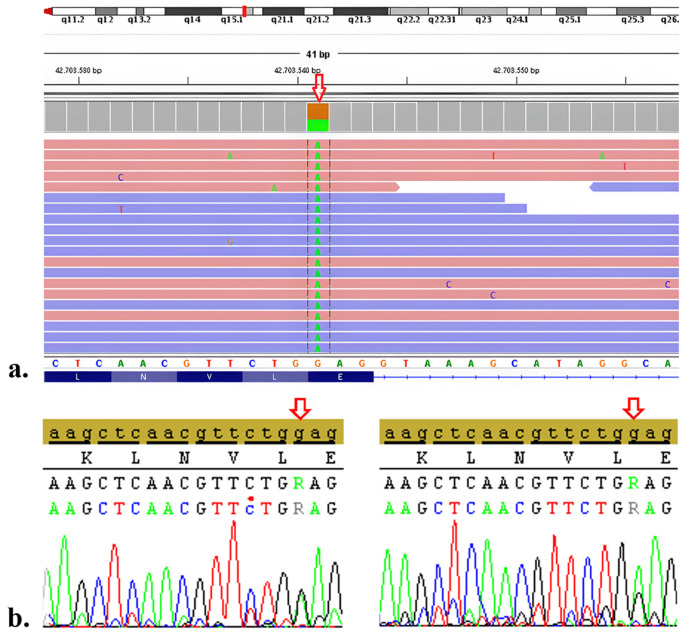
Next-generation sequencing result of CAPN3 variant of F1 (a), Sanger sequence analysis result of F1 (b), Sanger sequence analysis result of F1’s mother (c).

The molecular analysis results for all probands in this study are summarized in Table 2. In the case of F1, NGS analysis revealed the presence of the heterozygous variant NM_000070.3:c.2437G>A. Following the 2015 ACMG guidelines, the c.2437G>A variant was classified as VUS. To the best of our knowledge, this missense variant has not been reported in the literature previously. The variant was further confirmed through Sanger sequencing analysis, as it had not been reported before (Figure 1a). Additionally, this variant was not found in the gnomAD database.

For the segregation study, the proband’s mother, father, three children, and spouse underwent Sanger sequencing analysis. The heterozygous variant c.2437G>A was identified only in the mother, as per the analysis (Figure 1b). For F2, the results revealed a homozygous nonsense variant NM_000070.3:c.1118G>A, classified as pathogenic. In the case of F3, compound heterozygous variants were identified as NM_000070.3:c.550del and NM_000070.3:c.1303G>A, with frameshift and missense types, categorized as pathogenic and likely pathogenic, respectively. F4 exhibited a homozygous intronic variant, NM_000070.3:c.1745+4_1745+7del. This mutation occurred in close proximity to the splice donor region, and its clinical significance was reported as VUS. In the results for F5, a homozygous missense variant NM_000070.3:c.1342C>T was identified and classified as pathogenic. Both F6-I and F6-II carried the same homozygous missense variant NM_000070.3:c.1622G>A, reported as likely pathogenic.

### 3.4. In silico analysis results

**Figure 2:**
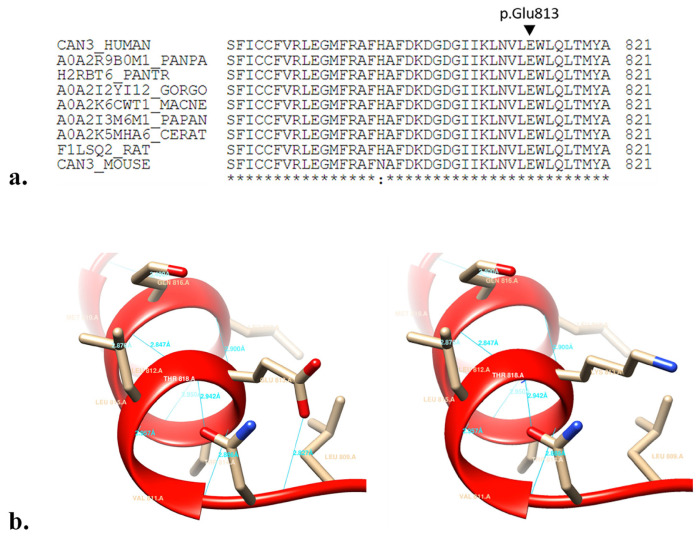
The figure shows that residue 813 of the CAPN3 protein is highly conserved in multiple alignments of different species (a). 3D visualization of the effect of the p.Glu813Lys mutation on the CAPN3 protein via the USCH Chimera software (b).

The variant c.2437G>A has not been previously reported in any database. This variant was generated by substituting the highly conserved polar acidic amino acid (glutamic acid) at position 813 in domain IV with a polar basic amino acid (lysine) (Figure 2a). Tools for predicting the clinical significance of variant c.2437G>A, as summarized in Table 3, consistently predicted the variant to be pathogenic. In-depth 3D visualization aimed at better understanding the structural effect of the p.(Glu813Lys) variant revealed that the mutation at residue 813 led to the loss of the hydrogen bond between Glu813 and Trp814. However, it was observed that the tertiary structure of the protein did not change (Figure 2b).

### 3.5. *CAPN3* variant characterization in Turkish vs global calpainopathy cohorts

**Figure 3:**
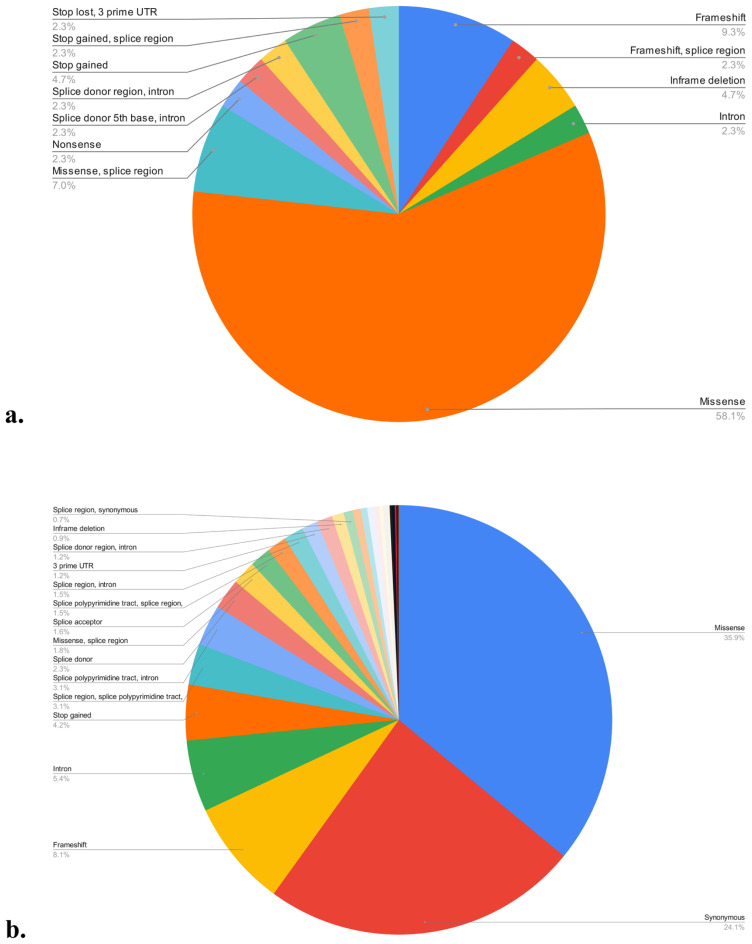
Turkish calpainopathy cohort CAPN3 mutation consequences (a) and global calpainopathy cohort CAPN3 mutation consequences (b). UTR, untranslated region.

**Figure 4:**
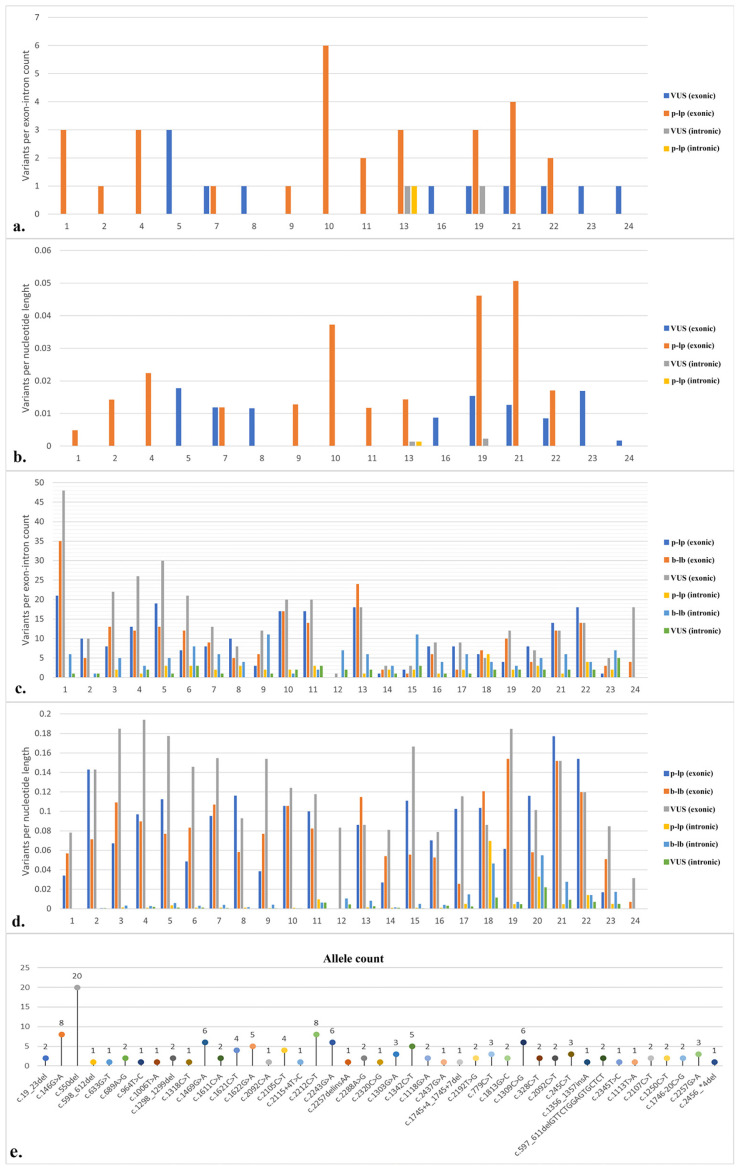
Variant distribution in the structure of CAPN3 (24 exons and 23 introns) and distribution by nucleotide length in the Turkish (a–b) and global (c–d) calpainopathy populations. CAPN3 variant allele count in the Turkish calpainopathy cohort (e). p, pathogenic; lp, likely pathogenic; VUS, variant of unknown significance; b, benign; lb, likely benign.

In the Turkish population, a total of 43 variants were identified, while in the global population, there were 1006 variants. Among the 43 Turkish variants, the most common consequence was missense, accounting for 58.1%, followed by frameshift at 9.3%, and missense in the splice region at 7% (Figure 3a). In contrast, among the 1006 global variants, missense was the most prevalent, comprising 35.9%, followed by synonymous at 24.1%, and frameshift at 8.1% (Figure 3b). In the global population, the consequences of variants were distributed as follows: 38% VUS, 35% benign and likely benign, and 27% pathogenic and likely pathogenic. In the Turkish population, consequences were 30% VUS and 70% pathogenic and likely pathogenic, with no benign variants observed. In the Turkish population, exon 10 had the highest frequency of pathogenic and likely pathogenic variants, while exon 5 had the highest frequency of VUS variants (Figure 4a). A likely pathogenic variant and a VUS variant were both present in intron 13, and a VUS variant was present in intron 19. When the average risk was calculated based on length, exon 21 had the highest risk, on average, in terms of pathogenic and likely pathogenic variants, whereas exon 5 had the highest risk for VUS variants (Figure 4b). In the global population, pathogenic and likely pathogenic, VUS, and benign variants were most frequently found in exon 1. For intronic variants, intron 18 was most common for pathogenic and likely pathogenic variants, intron 23 for VUS variants, and introns 9 and 15 for benign and likely benign variants (Figure 4c). In the global population, exon 21 had the highest mean risk by length for pathogenic and likely pathogenic variants, exon 4 for VUS variants, and exon 19 for benign variants (Figure 4d). Among intronic variants, the risk was greatest in intron 18 for pathogenic and likely pathogenic variants, and intron 20 for VUS and benign and likely benign variants (Figure 4d). For Turkish patients with calpainopathy, c.550del in exon 4 emerged as a hotspot variant, followed by c.2212C>T in exon 21 (Figure 4e). Statistical analysis of the distribution of variants across domains in terms of clinical significance suggested a random distribution. However, when considering all variants, 73% were found in the catalytic domain, 9% in EF-hand 3, 7% in EF-hand 1, 7% in EF-hand 2, and 5% in EF-hand 4. Calculating this distribution based on the length of the domains revealed 25% in EF-hand 4, 21% in EF-hand 2, 20% in EF-hand 1, 19% in catalytic, and 15% in EF-hand 3.

## 4. Discussion

We present the clinical and molecular results of 7 patients with calpainopathy in Türkiye. Additionally, we report a novel *CAPN3* variant associated with LGMDD1. The study cohort included patients with one heterozygous (F1), one compound heterozygous (F3), one homozygous intronic (F4), and three homozygous exonic (F2, F5, F6-I, F6-II) mutations. The common initial symptom of weakness in the proximal lower extremities was observed in all individuals except for F6-II. With the exception of F1, all patients were ambulant at the time of the evaluation.

In LGMDD1, affected individuals inherit a pathogenic *CAPN3* variant from the heterozygous parent [5]. While it is generally believed that LGMDD1 has a later onset and milder progression [4], there is evidence of early onset and clinical heterogeneity in this form, similar to LGMDR1 [22]. In our study, the individual with the c.2437G>A variant experienced initial symptoms in adolescence, presenting as muscle weakness, and became wheelchair-dependent approximately 16 years after onset. However, the patient did not exhibit calf hypertrophy or contracture. The family segregation study also demonstrated that the proband inherited this allele from the mother. Interestingly, the proband’s mother had no active complaints. It has been reported that parents carrying the heterozygous allele in LGMDD1 may not exhibit clinical features of the disease [4]. Recessive or dominant forms of calpainopathy exhibit significant clinical heterogeneity, even within families [4,23]. Prior to molecular analysis, the proband’s muscle biopsy was performed with the suspicion of sarcoglycanopathy, revealing a decrease in γ and δ sarcoglycan levels. However, no mutations were detected in the Sarcoglycan Gamma (*SGCG*) and Sarcoglycan Delta (*SGCD*) genes that encode these proteins in the subsequent molecular analysis. Sarcoglycan deficiency has been reported previously in a case with the *CAPN3* mutation [24]. Filamin C (FLNC), considered one of the substrates of CAPN3, is among the muscle-specific proteins that regulate muscle cells. FLNC regulates sarcoglycan by binding to γ and δ sarcoglycans from its C terminus [25]. CAPN3 cleaves the C-terminus of FLNC, disrupting the interaction between FLNC and γ and δ sarcoglycans [25]. The mutation identified in this case occurred in exon 23 in the PEF domain, which is the homodimerization and Ca2+ binding site of the CAPN3 protein [26]. The presence of Ca^2+^ is mandatory for CAPN3 activation. Upon Ca^2+^ binding, CAPN3 undergoes significant conformational changes to perform its proteolytic and nonproteolytic functions [27]. Therefore, the mutation in this region may have disrupted the interaction of CAPN3 with FLNC, potentially leading to decreases in sarcoglycan levels. The severe prognosis observed, similar to the recessive form in LGMDD1, which is typically expected to be milder and late-onset, might be attributed to sarcoglycan deficiency at the protein level. Additionally, bioinformatics tools indicated that this mutation resulted in the removal of the hydrogen bond between residues 813 and 814 of the protein, classifying the variant as pathogenic. Therefore, we think that the variant, not previously reported in the literature, exerts a dominant negative effect, consistent with the LGMDD1 phenotype. However, a more comprehensive analysis, including whole-exome sequencing or whole-genome sequencing, is required to exclude the effect of other defective genes.

The pathogenic variant c.1118G>A, located in exon 9 and resulting in a premature stop codon, has been previously associated with calpainopathy [28]. Indeed, the phenotype observed in F2, carrying this variant as homozygous in our cohort, was consistent with calpainopathy. F2 exhibited initial symptoms in the prepubertal period, presenting as weakness in the proximal lower extremities. With a high CK level, contractures, and increasing muscle weakness, F2 was diagnosed with moderate calpainopathy. Interestingly, the muscle biopsy result revealed partial or total negative dystrophin and β-dystroglycan levels, despite a positive CAPN3 staining result. The muscle biopsy result raised suspicion of Becker muscular dystrophy, leading to the analysis of F2’s Dystrophin gene through a multiplex ligation-dependent probe amplification method. However, no deletion or duplication was found in this gene. In approximately 80% of cases with calpainopathy, the CAPN3 protein is observed as reduced or absent in immunoblot or immunohistochemical (IHC) tests [29–31]. However, contrasting studies report that normal CAPN3 protein levels are found in patients diagnosed with calpainopathy at the genetic level [32,33]. Although IHC or immunoblotting has an important place in diagnosis, the presence of nonfunctional proteins at quantitatively normal levels reveals that genetic testing is the gold standard. A mutated CAPN3 gene showing normal levels of the protein product may indicate that this protein has lost its autolytic function due to the mutation [32]. Secondary dysferlin loss in calpainopathy has been reported previously [34]. To the best of our knowledge, secondary β-dystroglycan loss in calpainopathy has never been reported. The CAPN3 protein interacts with AHNAK in the dysferlin complex to maintain muscle membrane homeostasis [35]. Therefore, the secondary decrease in dysferlin in F2 would not be surprising. The relationship between CAPN3 and β-dystroglycan, on the other hand, is unknown. More research is needed to explain this secondary loss of β-dystroglycan.

F3, with the compound heterozygous variant, exhibited a clinical presentation consistent with calpainopathy. Onset symptoms in F3 included proximal muscle weakness in young adulthood, moderate progression, and a high CK level. Subsequently, myalgia complaints started. One of the variants, c.550del in exon 4, is classified as pathogenic and stands as the most common variant in Türkiye and Europe [3,36]. The other variant, c.1303G>A in exon 10, has been reported in the literature [37] and is classified as pathogenic/likely pathogenic according to ClinVar 4. Compound heterozygous cases, where this variant coexisted with a pathogenic variant, have been previously reported [37,38]. However, the muscle biopsy revealed only minor changes in a few areas.

The variant c.1745+4_1745+7del has been previously reported in the literature [39]. F4, who developed symptoms earliest in our cohort, carried this variant. Although the clinical significance of the variant was reported as VUS or likely pathogenic, F4 exhibited a phenotype consistent with calpainopathy. F4 presented calpainopathy features, including weakness in proximal limb muscles, calf hypertrophy, a high CK level, and muscle structure showing a dystrophic pattern. According to ClinVar^5^, the variant c.1745+4_1745+7del has conflicting interpretations, being classified as both likely pathogenic and VUS. This mutation, which occurred close to the splice_donor region, has the potential to affect the alternative splicing mechanism.

Muscle weakness progressed after Achilles tendon surgery in F5 with the homozygous variant c.1342C>T. F5 presented weakness in proximal limb muscles, a high CK level, contracture, and muscle structure with a dystrophic pattern. This variant is classified as pathogenic by ClinVar and has been associated with calpainopathy [40]. Numerous calpainopathy cases with this variant in exon 10 have been reported as homozygous and compound heterozygous [38,40,41].

The variant c.1622G>A in F6-I and F6-II has been previously reported in Türkiye [3]. This variant is classified as pathogenic/likely pathogenic on ClinVar. Located in exon 13, this variant has a mild effect on splicing, allowing the expression of normal transcripts but skipping a complete exon, resulting in the formation of an abnormally spliced transcript [42]. Although the siblings had the same homozygous missense variant, F6-I had more severe and earlier onset symptoms than F6-II. In the initial symptoms of F6-I, in addition to weakness in the proximal lower extremities, there was a complaint of toe walking, and its progression was moderate. However, F6-II showed a mild course with only a wobbly gait complaint, and no increase was observed in the complaint in the following periods. Indeed, due to the heterogeneous phenotype of calpainopathy, even siblings with the same variant may have different phenotypes [23]. The penetrance of calpainopathy is almost complete in adulthood, although some individuals may be asymptomatic until adulthood. As a result, the symptoms of F6-II, which currently only include a wobbly gait, may change in adulthood.

In the Turkish and global calpainopathy cohorts, missense mutations were the most common variants associated with calpainopathy in the *CAPN3* gene. This result is consistent with the LOVD and ClinVar databases. The most recent study revealing the frequency of variants in the Turkish population was conducted in 2006, and the c.550del variant was reported to be the most common [3]. Our study, conducted as of 2022, reaffirms that c.550del is the most common variant in the Turkish population. c.550del is also the most frequent variant in the European population [36]. Exon 10 is noteworthy for having the highest frequency of pathogenic and likely pathogenic variants in the Turkish population, while exon 1 holds this distinction for the global population. Exon 1 also has the highest frequency of benign and VUS variants. This is not surprising, as exon 1 is the longest one. Although there is no pathogenic single nucleotide variant in exon 24 in the global population, the only disease-related variant is c.2134_*219del, which includes exons 20–24. Additionally, exon 12 contained a single variant, which is VUS. Exon 21 had the highest risk of pathogenicity in both populations based on the calculation of variants per nucleotide. A study showing the exons with the highest average risk on a global scale indicated exons 15, 22, 21, 10, and 19 [36]. The introns 18 and 20 contained the highest frequency of both pathogenic and benign variants, according to estimates per nucleotide. Therefore, regardless of the consequences, introns 18 and 20 may be more susceptible to mutating. However, introns 18 and 20, the shortest introns of the *CAPN3* gene, are smaller than 100 bp, which may have contributed to the high frequency. The distribution of variants on CAPN3 domains did not differ significantly in terms of consequences in both populations. The distribution by length of domains showed that variants were most common in EF-hand domains. The EF-hand domain has calcium and metal binding properties, and especially calcium dysregulation plays an important role in the pathophysiology of calpainopathy [43]. The most common variant in Türkiye, c.550del, and the most frequently mutated exon 10 are located in the catalytic domain. This domain provides the cysteine-type peptidase, hydrolase, and peptidase activity of CAPN3. In the context of personalized therapy and diagnostic approaches, it is important that variants show different frequencies in gene structure and domains in distinct ethnic groups. Future research can, therefore, be done by taking into account these distributions. It is also noticeable that VUS is the most frequent consequence in the global population. This suggests that further work is required to provide VUS variants with definite clinical significance.

In conclusion, this retrospective study reviewed the molecular and clinical outcomes of all patients based on their previous medical records, to the extent they were available. Thus, the study presents a novel variant responsible for LGMDD1. The success of the NGS method in diagnosing calpainopathy, which presents a heterogeneous phenotype, is quite remarkable considering that muscle biopsy results can be misleading. In addition, the study analyzed the variants of Turkish and worldwide patients with calpainopathy and revealed the characteristics of the *CAPN3* gene comparatively. The results showed that the most frequent variant was c.550del, and the most mutations occurred at exon 10 in the Turkish cohort. Given this information, it can be concluded that it is crucial to take ethnic origins into account while developing precise gene therapy strategies.

## Supplementary Information



## Figures and Tables

**Table 1:** Clinical and laboratory outcomes of patients.

Family/patient	Sex	Onset (years)	Examination age (years)	Initial symptoms	Examination symptoms	Ambulant	Contracture	Calf hypertrophy	CK level (U/l)	Muscle biopsy
F1	F	14	30	Proximal lower limb weakness, myalgia	Progression of muscle weakness, bilateral deltoid muscles weakness	No	No	No	267	Dystrophic pattern, decreased γ and δ sarcoglycan levels
F2	M	9	12	Proximal lower limb weakness	Progression of muscle weakness	Yes	Yes	No	5935	Slightly changed fiber size, CAPN3 was positive, dystrophin and β dystroglycan levels were partial-total negatives
F3	M	20	24	Proximal lower limb weakness	Progression of lower and upper muscle weakness, myalgia	Yes	NA	Yes	1172	Slightly increased fiber size, increased nuclei, atrophic cells
F4	M	7	12	Proximal lower limb weakness	Progression of muscle weakness	Yes	NA	Yes	3080	Dystrophic pattern, increased endomysial connective tissue
F5	M	16	25	Proximal lower limb weakness, shortening of Achilles tendon	Progression of muscle weakness, hyperlordosis	Yes	Yes	NA	4267	Dystrophic pattern
F6-I	M	10	18	Proximal lower limb weakness, toe-walking, getting tired quickly	Progression of muscle weakness, hyperlordosis	Yes	Yes	NA	161	Dystrophic pattern
F6-II	M	14	16	Waddling walking	No progression	Yes	No	No	5397	Dystrophic pattern

NA: Not available

**Table 2:** Molecular analysis results of patients.

Family/patient	Variant	Exon	Protein change	Type of mutation	Zygosity	Clinical Significance
F1	c.2437G>A	23	p.(Glu813Lys)	Missense	Heterozygous	VUS
F2	c.1118G>A	9	p.(Trp373Ter)	Nonsense	Homozygous	P
F3	c.550del	4	p.(Thr184Argfs*36)	Frameshift	Heterozygous	P
	c.1303G>A	10	p.(Glu435Lys)	Missense	Heterozygous	LP
F4	c.1745+4_1745+7del	-	-	Splice_donor_+4	Homozygous	VUS
F5	c.1342C>T	10	p.(Arg448Cys)	Missense	Homozygous	P
F6-I	c.1622G>A	13	p.(Arg541Gln)	Missense	Homozygous	LP
F6-II	c.1622G>A	13	p.(Arg541Gln)	Missense	Homozygous	LP

*CAPN3* gene transcript ID: NM_000070.3; CAPN3 protein ID: NP_000061.1, P: pathogenic, LP: likely pathogenic, VUS: variant of unknown significance

**Table 3:** Predictions of the clinical significance of the novel NM_000070.3:c.2437G>A mutation.

BayesDel addAF prediction (rankscore)	BayesDel NoAF prediction (rankscore)	LoFtool prediction (score)	CADD Phred prediction (score/rankscore)	ClinPred prediction (rankscore)	PROVEAN prediction (rankscore)	MUTATION TASTER prediction (rankscore)	PolyPhen-2 prediction (HUMDIV score)
Damaging (0.89262)	Damaging (0.89126)	Probably damaging (0.0198)	Likely Deleterious (33/0.93179)	Damaging (0.69215)	Damaging (0.5663)	Disease-causing (0.81001)	Probably damaging (1.000)

## Data Availability

The data supporting the findings of this study are available from Erciyes University Faculty of Medicine Hospital, but restrictions apply to the availability of these data, which were used under license for the current study, and so are not publicly available. Data are, however, available from the authors upon reasonable request and with permission of Erciyes University Faculty of Medicine Hospital.

## Footnotes

1 Orphanet: Calpain 3 related limb girdle muscular dystrophy R1 [online]. Website https://www.orpha.net/consor/cgi-bin/OC_Exp.php?Lng=GB&Expert=267.0 [accessed 23 Nov 2022]

2 OMIM Entry - # 253600 - MUSCULAR DYSTROPHY, LIMB-GIRDLE, AUTOSOMAL RECESSIVE 1; LGMDR1 [online]. Website https://omim.org/entry/253600 [accessed 23 Nov 2022]

3 Graphs and statistics for the CAPN3 gene - Global Variome shared LOVD [online]. Website https://databases.lovd.nl/shared/genes/CAPN3/graphs [accessed 23 Nov 2022].

4 VCV000282173.16 - ClinVar – NCBI [online]. Website https://www.ncbi.nlm.nih.gov/clinvar/variation/282173/[accessed 29 Jul 2022]

5 VCV000194149.7 - ClinVar - NCBI. Website https://www.ncbi.nlm.nih.gov/clinvar/variation/194149/?new_evidence=false [accessed 24 Dec 2021]

## Abbreviations

3D: Three-dimensional
ACMG: American College of Medical Genetics and Genomics
Ca^2+^: Calcium ion
CAPN3: Calpain 3
CBSW: Calpain-type sandwich
CK: Creatine kinase
FLNC: Filamin-C
IHC: Immunohistochemical
IS: Insertion sequence
LGMDD1: Limb-girdle muscular dystrophy type dominant 1
LGMDR1: Limb-girdle muscular dystrophy type recessive 1
LOVD: Leiden Open Database of Variation
NGS: Next-generation sequencing
NS: N-terminus
PC: Protease core domain
PEF: Penta-E helix F helix
SGCD: Sarcoglycan Delta
SGCG: Sarcoglycan Gamma
VUS: Variant of unknown significance
