# Corrigendum to “A retrospective study on the clinical and molecular outcomes of calpainopathy in a Turkish patient cohort” [Turkish Journal of Medical Sciences 54 (1) 2024 86–98]

**DOI:** 10.55730/1300-0144.5861

**Published:** 2024-09-30

**Authors:** İzem Olcay ŞAHİN, Emine KARATAŞ, Mikail DEMİR, Büşra TAN, Hüseyin PER, Yusuf ÖZKUL, Munis DÜNDAR

**Affiliations:** 1 Department of Medical Genetics, Faculty of Medicine, Erciyes University, Kayseri, Turkiye; 2 Department of Pediatric Neurology, Faculty of Medicine, Children’s Hospital, Erciyes University, Kayseri, Turkiye

This corrigendum is written to correct an error in our previous publication. The term “LGMDD4” was incorrectly spelled as “LGMDD1” throughout the article. The correct term should be “LGMDD4”.

The authors apologize for any inconvenience caused and state that the scientific conclusions are unaffected.

